# Phenolic Constituents of Medicinal Plants with Activity against *Trypanosoma brucei*

**DOI:** 10.3390/molecules21040480

**Published:** 2016-04-12

**Authors:** Ya Nan Sun, Joo Hwan No, Ga Young Lee, Wei Li, Seo Young Yang, Gyongseon Yang, Thomas J. Schmidt, Jong Seong Kang, Young Ho Kim

**Affiliations:** 1 College of Pharmacy, Chungnam National University, Daejeon 305-764, Korea; yanansun@163.com (Y.N.S.); lakasis@lycos.co.kr (G.Y.L.); syyang@cnu.ac.kr (S.Y.Y.); 2 Leishmania Research Laboratory, Institut Pasteur Korea, 696 Sampyeong-dong, Bundang-gu, Seongnam-si, Gyeonggi 463-400, Korea; joohwan.no@ip-korea.org (J.H.N.); gyongseon.yang@ip-korea.org (G.Y.); 3 School of Biotechnology, Yeungnam University, Gyeongsan, Gyeongbuk 712-749, Korea; hasaegawa@ynu.ac.kr; 4 Institute of Pharmaceutical Biology and Phytochemistry (IPBP), University of Münster, PharmaCampus, Corrensstrasse 48, D-48149 Münster, Germany

**Keywords:** phenolic constituents, medicinal plants, *Trypanosoma brucei*, neglected tropical diseases, QSAR

## Abstract

Neglected tropical diseases (NTDs) affect over one billion people all over the world. These diseases are classified as neglected because they impact populations in areas with poor financial conditions and hence do not attract sufficient research investment. Human African Trypanosomiasis (HAT or sleeping sickness), caused by the parasite *Trypanosoma brucei*, is one of the NTDs. The current therapeutic interventions for *T. brucei* infections often have toxic side effects or require hospitalization so that they are not available in the rural environments where HAT occurs. Furthermore, parasite resistance is increasing, so that there is an urgent need to identify novel lead compounds against this infection. Recognizing the wide structural diversity of natural products, we desired to explore and identify novel antitrypanosomal chemotypes from a collection of natural products obtained from plants. In this study, 440 pure compounds from various medicinal plants were tested against *T. brucei* by in a screening using whole cell *in vitro* assays. As the result, twenty-two phenolic compounds exhibited potent activity against cultures of *T. brucei*. Among them, eight compounds—**4**, **7**, **11**, **14**, **15**, **18**, **20**, and **21**—showed inhibitory activity against *T. brucei*, with IC_50_ values below 5 µM, ranging from 0.52 to 4.70 μM. Based on these results, we attempt to establish some general trends with respect to structure-activity relationships, which indicate that further investigation and optimization of these derivatives might enable the preparation of potentially useful compounds for treating HAT.

## 1. Introduction

Human African trypanosomiasis (HAT, sleeping sickness) is a disease occurring exclusively in sub-Saharan Africa [1,2,3,4], and caused by two subspecies of the protozoan parasite *Trypanosoma brucei*; *Trypanosoma brucei gambiense* in West and Central Africa and *Trypanosoma brucei rhodesiense* in East Africa [5,6,7,8]. The geographic restriction of *T. brucei* to sub-Saharan Africa is due to the occurrence of the insect vectors, tsetse flies (genus *Glossina*) which only occur in tropical Africa. The two parasite subspecies produce different clinical forms of the disease. *T. b. gambiense* HAT takes a chronic course usually lasting for years, while *T. b. rhodesiense* HAT causes an acute course usually taking only months. Both forms inevitably lead to death if untreated [9,10]. Current clinical treatment relies on very few drugs, namely, suramin, pentamidine, melarsoprol and eflornithine (the latter now also in combination with nifurtimox) [11]. All of these present severe applicability limitations (e.g., suramin and pentamidin are only active in the early non-CNS stage of the disease, eflornithine is only effective against *T. b. gambiense* HAT and requires large amounts of drug for the treatment of a single patient). Severe toxic effects occur in many patients, especially in case of the arsenical drug melarsoprol which is still the only therapy for late stage *T. b. rhodesiense* HAT [11,12]. Moreover, the increase of drug resistant forms emphasize the urgent need for new drugs against HAT. The potential of natural products as lead compounds in drug development is well known and many natural products have shown activity against *T. brucei* and other parasites causing NTDs [13,14]. In the present communication we report on the results obtained by screening a library of 440 chemically diverse natural products obtained from medicinal plants against *T. brucei* leading to the discovery of a variety of compounds with significant anti-trypanosomal activity *in vitro*. Since a large fraction of these hits were phenolic in nature, we report here on these compounds and their antitrypanosomal activity. Hits of other chemical classes will be the subject of a subsequent communication.

## 2. Results and Discussion

### 2.1. Screening of Natural Product Library

**Figure 1:**
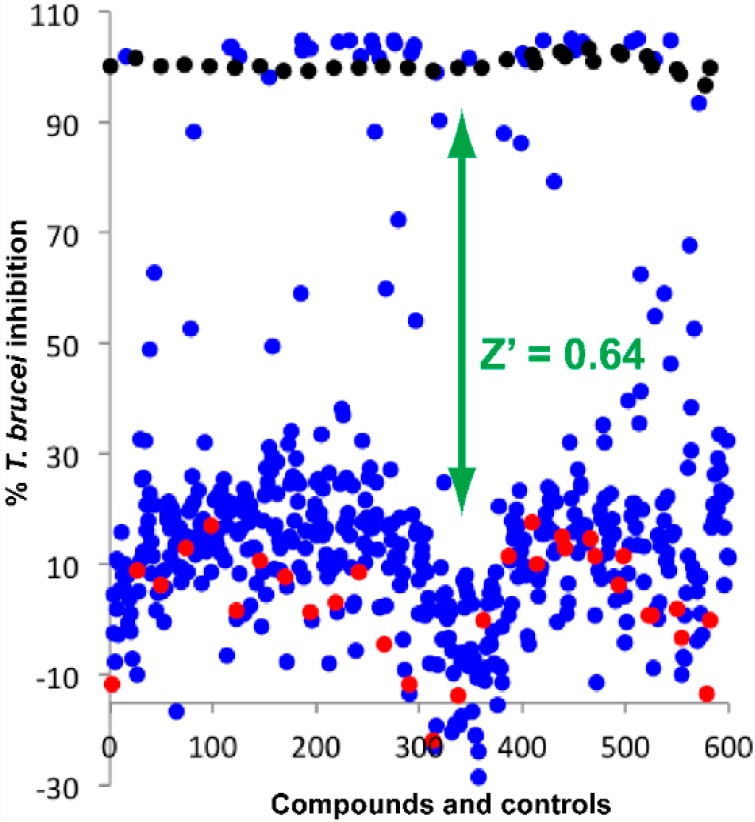
Screening of plant-derived natural products for inhibition of *T. brucei* growth: Distribution plot of the 440 natural compounds (**blue**), negative controls 0.5% DMSO (**red**), and positive controls (pentamidine) at IC_100_ (**black**).

To identify inhibitors of *T. brucei* growth, we conducted a screening of a proprietary library consisting of 440 purified natural products isolated from medicinal plants at the CNU laboratories. The main characteristics of this collection are summarized in Table S1 (Supplementary Materials). In total, 305 of the compounds complied with Lipinski’s rule of five. Of the 135 compounds presenting more than one violation, 33 violated two, 101 three and only one compound all four rules. Nevertheless, since, according to the 5th rule, natural products may also represent exceptions from the other rules, all compounds were submitted to antiparasitic screening performed at a single concentration of 5 ppm (µg/mL) of each purified natural product. Biological activity data were normalized using the growth inhibition of pentamidine (positive control) at 120 nM and that of DMSO 0.5% (negative control) as the maximum and minimum values. The Z′ factor for the controls was calculated to be 0.64 using the equation defined as Z′ = 1 − 3(σ_C_^+^ + σ_C_^−^)/|μ_C_^+^ − μ_C_^−^| where σ_C_^+^/σ_C_^−^ are the standard deviation (SD) of the positive/negative controls and μ_C_^+^/μ_C_^−^ are the mean values, indicating that the assay was performed with a sufficient window between the two controls (Figure 1).

**Figure 2:**
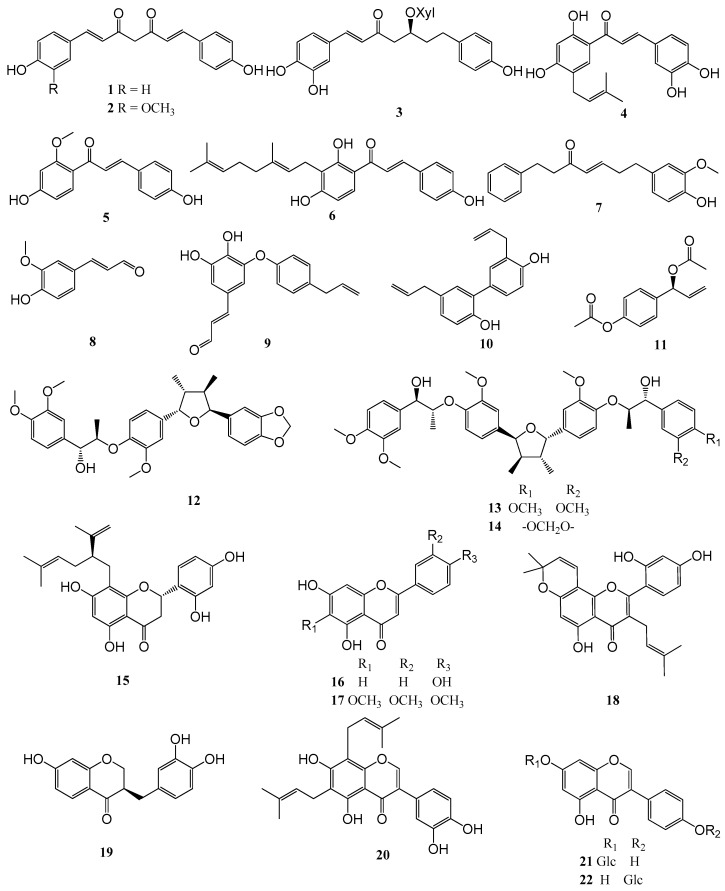
Structures of the 22 phenolic hit compounds with determined IC_50_ values.

Of the tested 440 compounds, 48 (11%) showed significant growth inhibition (GI ≥50%) and 32 strong inhibition (≥90%) of parasite growth at 5 ppm. Interestingly, 22 of these initial hits (compounds **1**–**22**, see Figure 2) were compounds with a phenolic structure, *i.e.*, had at least one free or substituted phenolic oxygen in their structure (alkaloids excluded). It is interesting to note that a relatively large fraction of the very active hits (69%) were of phenolic nature since the overall fraction of N-free phenolics in the library was only 51% (225 of 440 compounds). The 22 phenolic hit compounds were selected for a more detailed study of their activity against *T. brucei*.

### 2.2. Biological Activity of the Screening Compounds

Compounds **1**−**22** (*i.e*., bisdemethoxycurcumin (**1**), demethoxycurcumin (**2**), oregonin (**3**), broussochalcone A (**4**), 3-deoxysappanchalcone (**5**), xanthoangelol (**6**), 7-(4″-hydroxy-3″-methoxyphenyl)-1-phenylhept-4-en-3-one (**7**), 4-hydroxy-3-methoxycinnamaldehyde (**8**), obovatal (**9**), honokiol (**10**), 1′*S*-1′-acetoxychavicol acetate (**11**), saucerneol D (**12**), manassantin A (**13**), manassantin B (**14**), kushenol F (**15**), apigenin (**16**), eupatilin (**17**), morusin (**18**), 3-deoxysappanone B (**19**), 6,8-diprenylorobol (**20**), genistin (**21**), sophoricoside (**22**), see Figure 2) were selected for a more detailed determination of their inhibitory activity against *T. brucei*.

In order to determine IC_50_ values of these compounds, the dose dependent inhibitory activity against *T. brucei* cell growth was evaluated. As a result, the tested compounds showed significant anti-trypanosomal activities with IC_50_ values ranging from 0.52 to 16.42 μM (the data are summarized in Table 1; Dose-effect curves of all 22 compounds are reported as Figure S1, Supplementary Materials). Among the tested compounds, compounds **4**, **7**, **11**, **14**, **15**, **18**, **20**, and **21** showed potent inhibitory activity against *T. brucei*, with half maximal inhibitory concentration (IC_50_) values below 5 µM, respectively. Compounds **1**−**3**, **6**, **9**, **10**, **12**, **13**, **17**, **19**, and **22 **had weaker effects with IC_50_ values ranging between 5 and 10 µM. Compounds **5**, **8**, and **16**, in spite of presenting a high level of activity in the one-concentration screening, displayed only negligible effects with IC_50_ values greater than 10 µM. Some of these compounds (**1**, **2**, **3**, and **16**) had previously been reported to have anti-trypanosomal activity [15,16]. To further evaluate and select compounds with selective toxicity against *T. brucei*, we also tested the compounds’ effects on cell viability in human cells, namely, human embryonic kidney (HEK297T) and human hepatocellular carcinoma (HepG2) cell lines. The half maximal cytotoxicity concentration (CC_50_) was in no case determined since none of the compounds reached 50% inhibition of cell viability at the highest concentration tested. Therefore, exact selectivity indices [*s.i.* = CC_50_ (HEK293T or HepG2)/IC_50_ (*T. brucei*)] could not be determined but can be estimated to be larger than the cutoff value defined by the largest tested concentration in the cytotoxicity assay. All the results are reported in Table 1.

### 2.3. Structure-Activity Relationships

**Figure 3:**
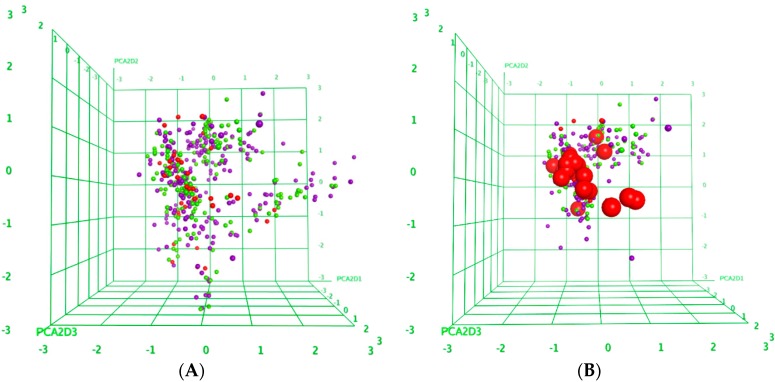
(**A**) Scores plots of a principal component analysis (PCA) peformed with 193 descriptors of 2D molecule structures of the 440 natural products tested. Positions of the compounds in the property space defined by the first three principal components PCA1-3 are color-coded: GI < 50%: Blue; 50% < GI < 90%: Yellow; GI > 90%: red; (**B**) Scores plot showing only phenolic compounds. Positions of the 22 phenolic hits highlighted by larger symbols.

The structures of the 440 tested natural products and the results from the biological screening were analyzed for general trends relating structure and antitrypanosomal activity. It should be noted that the activity data in this case are not suitable for a quantitative structure-activity relationship (QSAR) study since they are not on a molar scale and the data obtained only at one concentration do not necessarily represent a comparable point on the concentration effect curves. Nevertheless, 193 descriptors of 2D molecular structure were calculated using the Molecular Operations Environment software package (MOE [17]) and analyzed for overall trends to correlate with the percentage of growth inhibition observed at 5 ppm. To this end, a principal component analysis was performed with the descriptor matrix in order to capture the most important structural properties in a few latent variables. It was found that 84% of the variance encoded by the entire matrix of descriptors was explained by the first 10 principal components (PCs). None of these components showed a significant degree of correlation with the activity data. Figure 3A shows a 3D plot of the compounds’ positions (scores) on the first three principal component directions, which describe about 65% of the total variance in the descriptor matrix. The compounds are colored by activity, and it can be seen that there is no separation between the groups of very active (GI > 90%, red), active (GI between 50 and 90%, yellow) and weak or inactive compounds (GI < 50%, blue) in this 2D property space. A similar picture is seen when only the positions of compounds with phenolic groups are plotted (Figure 3B). In this plot, the positions of the highly active phenols chosen for IC_50_ determination are highlighted by larger symbols. It can be seen that most of them cluster in a relatively narrow volume of the overall structural space which means that they have relatively similar structural properties, which, however, are shared also by many inactive compounds.

**Figure 4:**
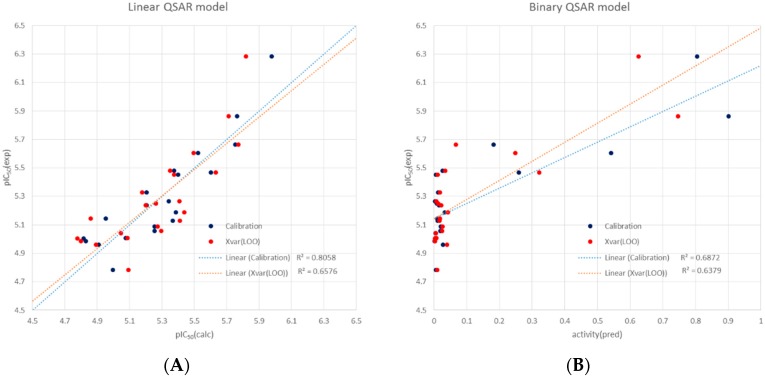
Performance of the linear (**A**) and binary (**B**) QSAR models: Plots of experimental *vs* calculated activity data. (pIC_50_ = −log (IC_50_ [M]). Calibration data are shown in blue, those for leave-one-out cross validation in red.

In order to investigate in more detail the activity differences observed between the 22 active compounds whose IC_50_ values were determined, a QSAR study was carried out. Molecular models of each structure generated with MOE were used to calculate a set of 95 descriptors related to properties of the three-dimensional structure (internal 3D descriptors), namely, ASA [17], q_frASA [18] and vsurf [18,19] descriptors. The descriptor matrix was submitted to variable selection using a genetic algorithm that is able to select the most relevant variables for multiple linear regression. It was found that a multiple linear model with four descriptors yielded a satisfactory and statistically significant model to describe the main features influencing the anti-trypanosomal activity in this group of phenolics. The regression equation is given by the following equation:

pIC_50_ = 35.08 − 0.58496 ASA- − 0.88538 vsurf_CW1 + 0.50298 vsurf_IW8 + 0.50627 ASAP6 (1)

where R2 = 0.81; RMSE = 0.15; Q2 = 0.66; RMSE (cross validated) = 0.20; *n* = 22; all variables were autoscaled, *i.e.*, descriptor values divided by their standard deviations. The resulting data obtained with this equation in the model calibration and cross validation are plotted in Figure 4A.

In a similar way, a binary QSAR model was also obtained with the same four descriptors that correctly discriminated between the more active (pIC_50_ > 5.5) and less active (pIC_50_ < 5.5) compounds (Figure 4B). The full statistical details of both models are reported in the Supplementary Materials.

The relevant descriptors are ASA-, the negatively charged part of the water-accessible surface area, ASAP6, the fraction of water-accessible surface area attributable to atoms with a positive partial charge between 0.25 and 0.30 e, vsurf_CW1, the first capacity factor (ratio of molecular interaction field surface calculated at −0.2 kcal/mol over total molecular surface) and vsurf_IW8, the hydrophilic integy moment calculated from the molecules’ interaction energies at the −6 kcal/mol level.

The negative regression coefficients of ASA- and vsuf_CW1 indicate that the properties related to these descriptors, *i.e.*, negatively charged surface area (mainly related to oxygen atoms and ratio of hydrophilic interactions over total molecular surface) have a detrimental effect on activity whereas the positive coefficients of vsurf_IW8 and ASAP6 indicate an enhancement of activity by larger values of these descriptors. In case of vsurf_IW8 this indicates that an uneven distribution of strongly interacting hydrophilic regions around the molecule or a concentration of such interactions in particular regions leads to an increased activity. ASAP6 is attributable to phenolic protons, *i.e.*, the accessible surface area related to such acidic protons. Thus, in combination with the other descriptors, free phenolic groups, possibly due to their H-bond donor function, are of positive influence on activity (it was observed that this descriptor could be substituted by the number of H-bond donor atoms which also yielded a positive regression coefficient; data not shown). Thus, the former two descriptors indicate an overall detrimental effect of hydrophilicity, the latter two point towards an overall enhancing effect of free phenolic hydrogen bond donors, best concentrated in a particular region of the molecule.

In order to investigate the QSAR models for a more general applicability, 45 phenolic compounds proven inactive in the initial screening (GI < 5%) were randomly chosen and also built as 3D models in the same way as the 22 major hits and their activities predicted from the respective descriptor values calculated in essentially the same manner. The linear model predicted for 37 of these compounds (82%) that the pIC_50_ should be <5.5 (IC_50_ > 3.2 µM) but only 22% (10 compounds) were predicted to have an IC_50_ > 10 µM. The binary model showed even somewhat better performance to predict correctly the inactivity of compounds since it returned relative activity values >0.5 only for 11% of the 45 inactives and only 2 of them (4.4%) were predicted to be really active with an activity >0.8.

These predictions show that the QSAR models derived from the more active phenolics perform reasonably well to predict low activity compounds (and can thus help to avoid testing many of them), even though the linear model tends to over-estimate the potency and predicted a moderate activity for a relatively large fraction of them.

A more qualitative discussion of the structure-activity relationships among the more active 22 compounds has to take into account that the structures of these phenolics are quite diverse so that details about contributions of particular structural features must remain somewhat speculative. Nevertheless, it is obvious that the three most active compounds, **20**, **15** and **4** are all prenylated flavonoid/chalcone derivatives. It is interesting to note that prenylated compounds of this type did not occur among the fully inactive phenolic molecules (GI < 50%) in the tested library so that this feature appears to be truly associated with activity. Nevertheless, it appears that prenylation is not a guarantee for high activity, since the geranylated chalcone **6** showed somewhat lower potency.

## 3. Materials and Methods 

### 3.1. Test Compounds

The tested compounds were isolated from the medicinal plants specified in Table 1 and were part of the in-house compound library of our laboratory (College of Pharmacy, Chungnam National University). These pure compounds were identified and assessed by 1D-NMR, HPLC and TLC analyses (purity in all cases >95%). Stock solutions in concentration 10.0 mM or 1000 ppm of the tested compounds were prepared in DMSO, kept at −20 °C, and diluted to the final concentration in fresh media before each experiment. In order to ensure unaffected cell growth, the final DMSO concentration did not exceed 0.5% in all experiments.

### 3.2. Chemicals, Reagents, and Media

Resazurin sodium salts, adenine, biotin, NaHCO_3_, phorbol 12-myristate 13-acetate, lectins PNA, paraformaldehyde (PFA) were purchased from Sigma (St. Louis, MO, USA); fetal bovine serum (FBS), HMI-9 medium, streptomycin/penicillin, HEPES, RPMI medium were purchased from Gibco (Grand Island, NY, USA); Draq5 was purchased from Biostatus (Leicestershire, UK); 384 well assay plates were purchased from Griener (Monroe, NC, USA).

### 3.3. Anti-Trypanosomal Activity on T. brucei

*T. b. brucei* strain 427 (bloodstream forms) were cultivated at 37 °C with 5% CO_2_ atmosphere in HMI-9 medium supplemented with 10% FBS. Cultivated parasites were seeded in 384 well plates (2500 parasite per well) and exposed to the natural compounds (5 ppm for the primary screening and two fold 10 point serial dilution starting from 5 ppm for the confirmatory assay) for 3 days of incubation. After the incubation, the parasites were exposed to 120 µM of resazurin sodium salt for 5 h. Then, the parasites were fixed with 4% PFA and the assay plates were read by Victor 3 (PerkinElmer, Waltham, MA, USA) at 530 nm_Ex_/590 nm_Em_. Pentamidine was used as a reference drug and DMSO 0.5% was used as a drug-negative control [20,21].

### 3.4. Cytotoxicity Assays

HEK293T and HepG2 cells were cultured at 37 °C with 5% CO_2_ in Dulbecco’s modified eagle medium containing 10% FBS. Both cell lines were plated individually in 384 well plates (4000 cells per well) and the natural compounds (two fold 10 points serial dilution starting from 5 ppm) were added for 3 days of incubation. After the incubation, 10.0 µL of a 280 µM solution of resazurin sodium salt in water was added to the cells in the assay plate for at least 5 h (final concentration, 40.0 µM of resazurin) and resazurin reduction was measured with a Victor 3™ fluorimeter (PerkinElmer) at 530 nm_Ex_/590 nm_Em_.

### 3.5. Molecular Modelling and QSAR analyses

All computational investiagations were performed with the molecular operations environment MOE [17]. The chemical structures of all 440 compounds were imported from mol-files generated with ChemDraw 12.0 into a MOE database. 193 2D descriptors available in MOE were generated for all compounds and a principal component analysis was carried out for the full data matrix, leading to the scores plots shown in Figure 3. Details of the PCA are reported in the Supplementary Materials.

For the 22 active hit compounds and for the randomly chosen 45 inactive phenolics, 3D molecular models were generated and geometry optimized by performing a low mode dynamics conformational search with standard settings. For each structure, the lowest energy conformer was used to calculate 3D molecular descriptors (ASA and vsurf descriptors). The fractional ASA descriptors (q_frASA) were calculated as reported earlier [18]. The vsurf descriptors are calculated by MOE according to literature [19].

QSAR modelling was performed by applying a genetic algorithm from MOE script GA.svl (available via the MOE svl exchange site) to the full descriptor matrix of the 22 active hits and limiting the number of variables in the models to 4. The resulting best linear regression model as reported in QSAR equation 1 was then used for further evaluation and for activity prediction of the 45 inactives. A binary QSAR model using the same four descriptors was also generated with the MOE/Quasar function and used for the same purposes. All molecular data are available from the corresponding author (TJS) on request.

## 4. Conclusions

The present search for new inhibitors of *T. brucei* growth, based on a screening of 440 compounds from medicinal plants, led to the identification of 32 natural products with high activity at 5 ppm concentration against bloodstream forms of this parasite. Of these, 22 were plant phenolics which were then investigated in more detail and four of them yielded IC_50_ values below 3 µM. None of the 22 hits displayed significant cytotoxicity against two human cell lines (HEK293T and HepG2; CC_50_ not reached at highest tested concentration). The remaining screening hits of non-phenolic nature will be subject of a subsequent report. QSAR modelling revealed several molecular features that enhance the antitrypanosomal activity within this series, as well as some that decrease the potency. The resulting linear and binary QSAR models may be used with some certainty to make activity predictions for further, yet untested plant phenolics. Overall, even though no extremely potent hits were found among these natural products, four representatives of the present compound set may be considered interesting starting points for further detailed investigations (activity against further strains of *T. brucei*, mechanism of action, *in vivo* activity) with the aim of optimizing the structures in order to develop new therapies against *T. brucei* infection.

## Figures and Tables

**Table 1:** *In* *vitro* antiparasitic and cytotoxic activities of 22 phenolic compounds from medicinal plants.

Compounds	*T. brucei* IC_50 _^a^ (µM)	Cytotoxicity ^a^ CC_50_ Values (µM)		*s.i* ^b^		Species
		HEK239T	HepG2	HEK239T	HepG2	
**1**	9.84 ± 0.84	>32.47	>32.47	>3.30	>3.30	*Curcuma longa*
**2**	7.19 ± 1.02	>21.35	>29.55	>2.97	>4.11	*Curcuma longa*
**3**	9.12 ± 0.63	>20.88	>20.88	>2.29	>2.29	*Alnus japonica*
**4**	2.17 ± 0.50	>28.81	>29.19	>13.28	>13.54	*Broussonetia papyrifera*
**5**	10.37 ± 3.36	>30.18	>37.12	>2.91	>3.58	*Caesalpinia sappan*
**6**	7.42 ± 1.12	>25.45	>25.45	>3.43	>3.43	Angelica keiskei
**7**	2.48 ± 0.02	>32.36	>32.36	>13.05	>13.05	*Alpinia officinarum*
**8**	16.42 ± 2.78	>48.11	>56.97	>2.93	>3.47	*Cinnamomum cassia*
**9**	9.89 ± 0.51	>33.82	>33.82	>3.42	>3.42	*Machilus thunbergii*
**10**	8.76 ± 0.38	>37.58	>37.58	>4.29	>4.29	*Machilus thunbergii*
**11**	4.70 ± 1.53	>23.22	>29.99	>4.94	>6.38	*Alpinia galanga*
**12**	5.61 ± 0.09	>16.21	>18.63	>2.89	>3.32	*Saururus chinensis*
**13**	5.43 ± 0.26	>11.84	>13.68	>2.18	>2.52	*Saururus chinensis*
**14**	3.54 ± 0.49	>11.04	>13.91	>3.12	>3.93	*Saururus chinensis*
**15**	1.37 ± 0.01	>23.76	>23.76	>17.34	>17.34	*Sophora flavescens*
**16**	10.96 ± 2.64	>37.04	>37.04	>3.38	>3.38	*Agrimonia pilosa*
**17**	8.14 ± 1.37	>29.14	>29.14	>3.58	>3.58	*Artemisia vulgaris*
**18**	3.40 ± 0.15	>23.73	>23.73	>6.98	>6.98	*Morus alba*
**19**	5.80 ± 0.07	>35.03	>35.03	>6.04	>6.04	*Caesalpinia sappan*
**20**	0.52 ± 0.01	>24.10	>24.10	>46.34	>46.34	*Cudrania tricuspidata*
**21**	3.31 ± 0.75	>23.13	>23.13	>6.99	>6.99	*Glycine max*
**22**	6.50 ± 0.52	>23.14	>23.14	>3.56	>3.56	*Sophora japonica*
Pentamidine ^c^	0.004 ± 0.003	<0.40	<0.40	<26.7	<26.7	-
Chlorpromazine ^c^	-	19.89	16.50	-	-	-

^a^ Selectivity relative to HEK293T and HepG2 cells for selected potent compounds; ^b^ *s.i.*: Selectivity index; ^c^ Positive control. All data represent the mean ± SD of at least three independent experiments performed in triplicates (*p* < 0.01).

## Supplementary Materials

Supplementary materials can be accessed at: http://www.mdpi.com/1420-3049/21/4/480/s1.
