# Supplementary material for: Phenolic Constituents of Medicinal Plants with Activity against Trypanosoma brucei
Source: Molecules. 2016 Apr 12;21(4):480. doi: 10.3390/molecules21040480 (PMC6273235; doi:10.3390/molecules21040480)
Supplement: Supplementary file 1 [file molecules-21-00480-s001.pdf]

Supplementary Materials: Phenolic Constituents of Medicinal Plants with Activity against *Trypanosoma brucei*

Ya Nan Sun, Joo Hwan No, Ga Young Lee, Wei Li, Seo Young Yang, Gyongseon Yang, Thomas J. Schmidt, Jong Seong Kang and Young Ho Kim

Table S1. Major characteristics of the compound library studied; number of unique compounds: 440.

	Max	Min	Mean	Median
%GI at 5 ppm	105.1	−28.4	20.56	15.40
molecular mass	1550	122	470.3	392.5
logP(ow) [a]	14.16	−9.29	1.88	2.21
logS [a]	0.714	−14.34	−4.22	−3.77
vdw_area (A2) [a]	1306.28	123.09	406.96	345.13
vdw_vol (A3) [a]	1535.57	143.85	512.28	439.56
TPSA [a]	611.58	0.00	137.04	99.26
n_acc [a]	37	0	8.05	6.00
n_don [a]	23	0	4.56	3.00
a_aro [a]	48	0	7.36	6.00
a_nC [a]	69	5	24.73	21.00
a_nO [a]	38	0	8.58	7.00
a_nN [a]	16	0	0.19	0.00
b_count [a]	230	15	68.68	55.50
b_single [a]	228	8	59.22	44.00
b_double [a]	14	0	2.02	2.00
b_triple [a]				
Lip_druglike [a]	1	0	0.69	1.00
Lip_violations [a]	4	0	1.00	0.00

[a] Descriptors calculated with MOE [14].

Table S2. 2D descriptors calculated for 440 test compounds and used in the PCA.

BCUT_PEOE_0	PEOE_VSA-1	SlogP_VSA0	b_count	vsa_acid
BCUT_PEOE_1	PEOE_VSA-2	SlogP_VSA1	b_double	vsa_base
BCUT_PEOE_2	PEOE_VSA-3	SlogP_VSA2	b_heavy	vsa_don
BCUT_PEOE_3	PEOE_VSA-4	SlogP_VSA3	b_max1len	vsa_hyd
BCUT_SLOGP_0	PEOE_VSA-5	SlogP_VSA4	b_rotN	vsa_other
BCUT_SLOGP_1	PEOE_VSA-6	SlogP_VSA5	b_rotR	vsa_pol
BCUT_SLOGP_2	PEOE_VSA_FHYD	SlogP_VSA6	b_single	weinerPath
BCUT_SLOGP_3	PEOE_VSA_FNEG	SlogP_VSA7	b_triple	weinerPol
BCUT_SMR_0	PEOE_VSA_FPNEG	SlogP_VSA8	balabanJ	zagreb
BCUT_SMR_1	PEOE_VSA_FPOL	SlogP_VSA9	bpol	
BCUT_SMR_2	PEOE_VSA_FPOS	TPSA	chi0	
BCUT_SMR_3	PEOE_VSA_FPPOS	VAdjEq	chi0_C	
FCharge	PEOE_VSA_HYD	VAdjMa	chi0v	
GCUT_PEOE_0	PEOE_VSA_NEG	VDistEq	chi0v_C	
GCUT_PEOE_1	PEOE_VSA_PNEG	VDistMa	chi1	
GCUT_PEOE_2	PEOE_VSA_POL	Weight	chi1_C	
GCUT_PEOE_3	PEOE_VSA_POS	a_IC	chi1v	
GCUT_SLOGP_0	PEOE_VSA_PPOS	a_ICM	chi1v_C	
GCUT_SLOGP_1	Q_PC+	a_acc	chiral	

GCUT_SLOGP_2	Q_PC-	a_acid	chiral_u
GCUT_SLOGP_3	Q_RPC+	a_aro	density
GCUT_SMR_0	Q_RPC-	a_base	diameter
GCUT_SMR_1	Q_VSA_FHYD	a_count	lip_acc
GCUT_SMR_2	Q_VSA_FNEG	a_don	lip_don
GCUT_SMR_3	Q_VSA_FPNEG	a_donacc	lip_druglike
Kier1	Q_VSA_FPOL	a_heavy	lip_violation
Kier2	Q_VSA_FPOS	a_hyd	logP(o/w)
Kier3	Q_VSA_FPPOS	a_nB	logS
KierA1	Q_VSA_HYD	a_nBr	mr
KierA2	Q_VSA_NEG	a_nC	mutagenic
KierA3	Q_VSA_PNEG	a_nCl	nmol
KierFlex	Q_VSA_POL	a_nF	opr_brigid
PC+	Q_VSA_POS	a_nH	opr_leadlike
PC-	Q_VSA_PPOS	a_nI	opr_nring
PEOE_PC+	RPC+	a_nN	opr_nrot
PEOE_PC-	RPC-	a_nO	opr_violation
PEOE_RPC+	SMR	a_nP	petitjean
PEOE_RPC-	SMR_VSA0	a_nS	petitjeanSC
PEOE_VSA+0	SMR_VSA1	apol	pos_fr_vsa
PEOE_VSA+1	SMR_VSA2	ast_fraglike	radius
PEOE_VSA+2	SMR_VSA3	ast_fraglike_ext	reactive
PEOE_VSA+3	SMR_VSA4	ast_violation	rings
PEOE_VSA+4	SMR_VSA5	ast_violation_ext	rsynth
PEOE_VSA+5	SMR_VSA6	b_1rotN	vdw_area
PEOE_VSA+6	SMR_VSA7	b_1rotR	vdw_vol
PEOE_VSA-0	SlogP	b_ar	vsa_acc

Table S3. 3D descriptors calculated for 22 active and 45 inactive compounds and used for QSAR.

ASA	vsurf_EWmin1	vsurf_Wp6
ASA+	vsurf_EWmin2	vsurf_Wp7
ASA-	vsurf_EWmin3	vsurf_Wp8
ASAN1	vsurf_G	
ASAN2	vsurf_HB1	
ASAN3	vsurf_HB2	
ASAN4	vsurf_HB3	
ASAN5	vsurf_HB4	
ASAN6	vsurf_HB5	
ASAN7	vsurf_HB6	
ASAP1	vsurf_HB7	
ASAP2	vsurf_HB8	
ASAP3	vsurf_HL1	
ASAP4	vsurf_HL2	
ASAP5	vsurf_ID1	
ASAP6	vsurf_ID2	
ASAP7	vsurf_ID3	
ASA_H	vsurf_ID4	
ASA_P	vsurf_ID5	
vsurf_A	vsurf_ID6	
vsurf_CP	vsurf_ID7	
vsurf_CW1	vsurf_ID8	
vsurf_CW2	vsurf_IW1	

vsurf_CW3	vsurf_IW2
vsurf_CW4	vsurf_IW3
vsurf_CW5	vsurf_IW4
vsurf_CW6	vsurf_IW5
vsurf_CW7	vsurf_IW6
vsurf_CW8	vsurf_IW7
vsurf_D1	vsurf_IW8
vsurf_D2	vsurf_R
vsurf_D3	vsurf_S
vsurf_D4	vsurf_V
vsurf_D5	vsurf_W1
vsurf_D6	vsurf_W2
vsurf_D7	vsurf_W3
vsurf_D8	vsurf_W4
vsurf_DD12	vsurf_W5
vsurf_DD13	vsurf_W6
vsurf_DD23	vsurf_W7
vsurf_DW12	vsurf_W8
vsurf_DW13	vsurf_Wp1
vsurf_DW23	vsurf_Wp2
vsurf_EDmin1	vsurf_Wp3
vsurf_EDmin2	vsurf_Wp4
vsurf_EDmin3	vsurf_Wp5

Table S4. Statistical details of linear QSAR model
QuaSAR-model(PLS): i:/documents/thomas schmidt/forschung/neglected_diseases/diverse_projekte/kim_kang_database/22 compounds for 3d structure-1st/22mols_for publication.mdb
Thu Mar 17 13:16:17 2016

Activity Field : pIC50 Tbr
Condition Limit : 1e+006
Component Limit : 0

Observations : 22
Descriptors : 4
Components Used : 4
Condition Number : 211829.86

ROOT MEAN SQUARE ERROR (RMSE): 0.14824
CORRELATION COEFFICIENT (R2) : 0.80580
CROSS-VALIDATED RMSE : 0.19818
CROSS-VALIDATED R2 : 0.65759

ESTIMATED LINEAR MODEL

pIC50 Tbr =
11.80063
-0.00393 * ASA-
+0.04325 * vsurf_IW8
+0.02142 * ASAP6
-2.18140 * vsurf_CW1

ESTIMATED NORMALIZED LINEAR MODEL (SD = Standard Deviation)

pIC50 Tbr / SD(pIC50 Tbr) =
35.08006
-0.58496 * ASA- / SD(ASA-)
+0.50298 * vsurf_IW8 / SD(vsurf_IW8)
+0.50627 * ASAP6 / SD(ASAP6)
-0.88538 * vsurf_CW1 / SD(vsurf_CW1)

RELATIVE IMPORTANCE OF DESCRIPTORS

0.660690 ASA-
0.568092 vsurf_IW8
0.571809 ASAP6
1.000000 vsurf_CW1

Table S5. Statistical details of binary QSAR model
QuaSAR-model(Binary): i:/documents/thomas schmidt/forschung/neglected_diseases/diverse_projekte/kim_kang_database/22 compounds for 3d structure-1st/22mols_for publication.mdb
Thu Mar 17 08:22:22 2016

Activity Field	: pIC50 Tbr	
Binary Threshold	: 5.5	
Smooth	: 0.25	
Condition Limit	: 1e+006	
Component Limit	: 0	
Active Observations	: 4	
Inactive Observations	: 18	
Observations	: 22	
Descriptors	: 4	
Components Used	: 4	
	QuaSAR Model	Chance
Total Accuracy	: 0.954545	0.731405
Significance (p-value)	: 1.820816e-002	
Accuracy on Active	: 0.750000	0.136364
Accuracy on Inactive	: 1.000000	0.863636
Significance (p-value)	: 1.349252e-003	

CROSS-VALIDATED STATISTICS

	QuaSAR Model	Chance
X-Validated Total Accuracy	: 0.909091	0.760331
Significance (p-value)	: 1.021492e-001	
X-Validated Accuracy on Active	: 0.500000	0.090909
X-Validated Accuracy on Inactive	: 1.000000	0.909091
Significance (p-value)	: 1.943558e-002	

COMPONENT DISTRIBUTION COMPARISON

comp	rmse	1-corr
----	------------	--------
1	3.162571	0.070774
2	3.261646	0.338644
3	3.196894	0.122045
4	3.292078	0.528840

DESCRIPTOR IMPORTANCE

0.525307 vsurf_CW1
0.329119 ASAP6
0.129864 vsurf_IW8
0.075632 ASA-

Table S6. Experimental pIC_{50} (-log IC_{50} [M]) values and data calculated in the model calibration (PRED) and leave-one-out cross validation (XPRED) of the linear and binary QSAR models.

Compound	pIC50 Tbr(exp)	PRED_linear	X_PRED_linear	PRED_binary	XPRED_binary
**20**	6.284	5.976	5.819	0.805	0.626
**15**	5.863	5.765	5.711	0.902	0.746
**4**	5.664	5.751	5.771	0.182	0.067
**7**	5.606	5.522	5.495	0.542	0.248
**21**	5.480	5.374	5.349	0.027	0.035
**18**	5.469	5.603	5.632	0.259	0.322
**14**	5.451	5.400	5.374	0.005	0.012
**11**	5.328	5.204	5.177	0.013	0.017
**13**	5.265	5.341	5.406	0.003	0.007
**12**	5.251	5.261	5.264	0.009	0.012
**19**	5.237	5.206	5.198	0.016	0.022
**22**	5.187	5.387	5.438	0.032	0.042
**2**	5.143	4.951	4.858	0.010	0.017
**6**	5.130	5.367	5.411	0.012	0.016
**17**	5.089	5.253	5.273	0.020	0.026
**10**	5.057	5.255	5.295	0.020	0.025
**3**	5.040	5.046	5.046	0.004	0.005
**1**	5.007	5.075	5.087	0.004	0.008
**9**	5.005	4.815	4.777	0.002	0.003
**5**	4.984	4.829	4.799	0.002	0.003
**16**	4.960	4.910	4.892	0.027	0.039
**8**	4.785	4.995	5.093	0.006	0.010

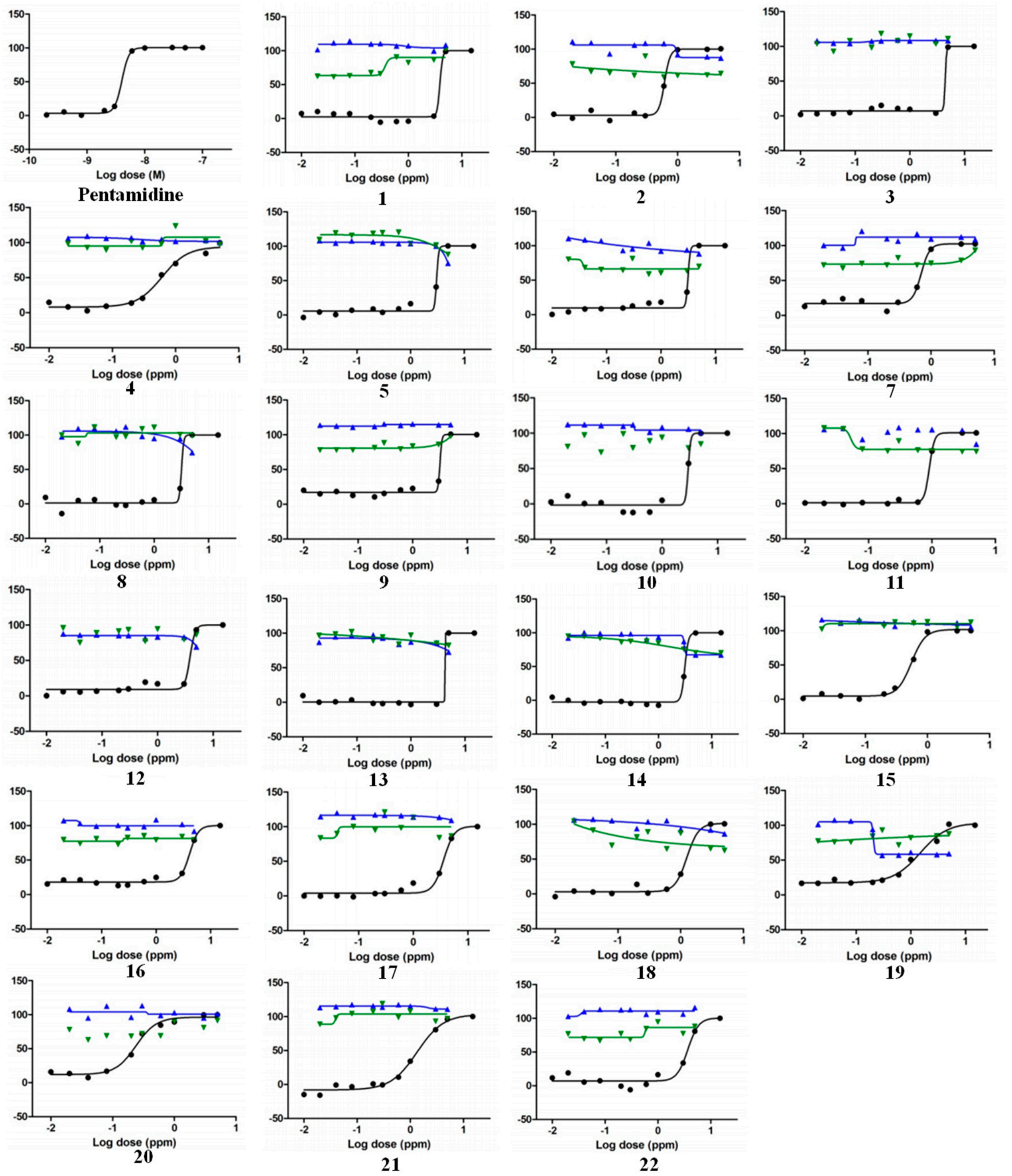

Figure S1. Dose-effect curves for anti-*T. brucei* (black) and cytotoxic activity on HEK293T (blue) and HepG2 (green) cells of compounds **1**–**22**. The vertical axis represents growth inhibitory activity in %, in relation to an untreated control.
